# The effect of common dental fixtures on treatment planning and delivery for head and neck intensity modulated proton therapy

**DOI:** 10.1002/acm2.13973

**Published:** 2023-03-27

**Authors:** Yue‐Houng Hu, Wan Chan Tseung Hok Seum, Ashley Hunzeker, Olivia Muller, Robert L. Foote, Daniel W. Mundy

**Affiliations:** ^1^ Department of Radiation Oncology Division of Medical Physics and Biophysics Brigham and Women's Hospital Dana‐Farber Cancer Institute, and Harvard Medical School Boston Massachusetts USA; ^2^ Department of Radiation Oncology Division of Medical Physics Mayo Clinic Rochester Minnesota USA; ^3^ Department of Advanced Prosthodontics Mayo Clinic Rochester Minnesota USA

**Keywords:** dental fixtures, dose perturbation, dosimetry, IMPT, Monte Carlo, proton therapy

## Abstract

**Purpose:**

Proton treatment plan perturbation by common dental fixtures such as amalgams (Am) and porcelain‐fused‐to‐metal (PFM) crowns has, to date, been uncharacterized. Previous studies have been conducted to determine the physical effect of these materials within the beam path for single spots, but their effects on complex treatment plans and clinical anatomy have not yet been quantified. The present manuscript aims to study the effect of Am and PFM fixtures on proton treatment planning in a clinical setting.

**Methods:**

An anthropomorphic phantom with removable tongue, maxilla, and mandible modules was simulated on a clinical computed tomography (CT) scanner. Spare maxilla modules were modified to include either a 1.5 mm depth central groove occlusal amalgam (Am) or a porcelain‐fused‐to‐metal (PFM) crown, implanted on the first right molar. Modified tongue modules were 3D printed to accommodate several axial or sagittal oriented pieces of EBT‐3 film. Clinically representative spot‐scanning proton plans were generated in Eclipse v.15.6 using the proton convolution superposition (PCS) algorithm v.15.6.06 using a multi‐field optimization (MFO) technique with the goal of delivering a uniform 54 Gy dose to a clinical target volume (CTV) typical of a base‐of‐tongue (BoT) treatment. A typical geometric beam arrangement of two anterior oblique (AO) beams and a posterior beam was employed. Plans optimized without any material overrides were delivered to the phantom A) without implants; B) with Am fixture; or C) with PFM crown. Plans were also reoptimized and delivered with inclusion of material overrides to equate relative stopping power of the fixture with that of a previously measured result.

**Results:**

Plans exhibit slightly greater dose weight towards AO beams. The optimizer accounted for inclusion of fixture overrides by increasing beam weights to the beam closest to the implant. Film measurements exhibited cold spots directly within the beam path through the fixture in plans with and without overridden materials. Cold spots were somewhat mitigated in plans including overridden materials in the structure set but were not entirely eliminated. Cold spots associated with Am and PFM fixtures were quantified at 17% and 14% for plans without overrides, respectively, and 11% and 9% with using Monte Carlo simulation. Compared with film measurements and Monte Carlo simulation, the treatment planning system underestimates the dose shadowing effect in plans including material overrides.

**Conclusions:**

Dental fixtures create a dose shadowing effect directly in line with the beam path through the material. This cold spot is partially mitigated by overriding the material to measured relative stopping powers. Due to uncertainties in modeling perturbation through the fixture, the magnitude of the cold spot is underestimated using the institutional TPS when compared to measurement and MC simulation.

## 1 INTRODUCTION

Intensity modulated proton therapy (IMPT) may provide outsized benefits in the treatment of head and neck (H&N) cancers because of the improved dose conformality afforded by the particle's finite range. Dental fixtures, commonplace in an aging population receiving radiation treatment, are as of yet uncharacterized for proton therapy beams in the context of complex shapes of the fixtures themselves and within the anatomy in which they reside. Typical clinical approaches involve some variation on an avoidance strategy: most commonly through adjustment of beam geometry, prevention of spot placement through the implants, or declination of treatment.

Attempts at characterizing treatments of patients with implanted dental fixtures have largely been limited to photon therapy treatment paradigms,^1^, ^2^, ^3^, ^4^, ^5^, ^6^, ^7^, ^8^, ^9^, ^10^, ^11^, ^12^, ^13^ with few addressing proton therapy specific concerns.^14^, ^15^ A prior study by this group has attempted to characterize the perturbation of single proton spots by a variety of dental fixture materials within the context of a simple phantom setup.^16^ The present study aims to expand upon the findings of the prior experiments by determining the effects of dose perturbation by those materials in clinically representative proton treatment plans delivered to an anthropomorphic phantom, where realistic fixtures are installed. Predicted doses from the institutional treatment planning system (TPS: Eclipse v.15.6 using the proton convolution superposition algorithm v.15.6.06) were measured using a film setup and also compared with previously validated Monte Carlo simulations.

## 2 METHODS

### 2.1 Phantom measurements

**FIGURE 1:**
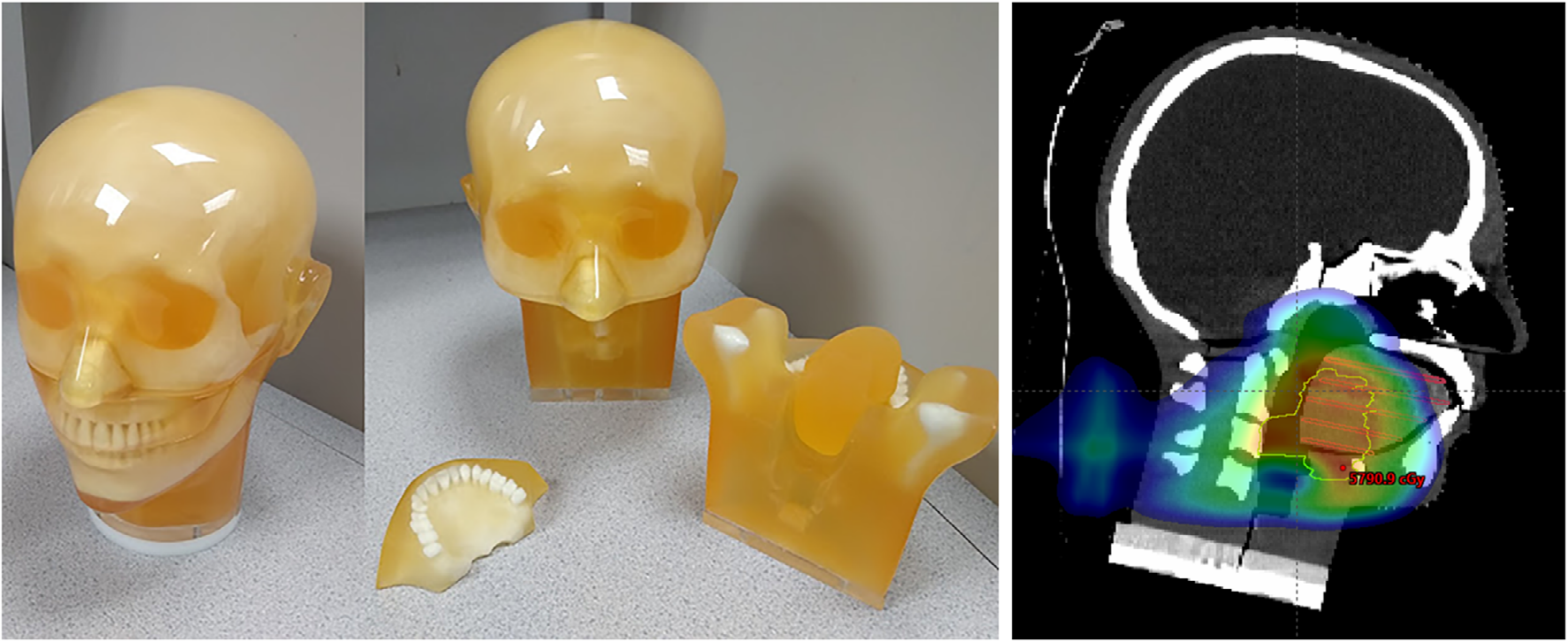
Photograph of the PH‐47 anthropomorphic dental radiology head phantom (left) along with a sagittal slice of the reconstructed simulation CT and treatment plan (right).

All measurements in the study were based on an anthropomorphic dental radiology head phantom (PH‐47; Kyoto Kagaku Co., LTD, Kyoto, Japan). The phantom (Figure 1, left) is composed of materials each formulated to model a specific tissue, achieving targeted HU numbers. A single sagittal slice of the reconstructed simulation CT and treatment plan (control) through the center of the prescribed target is also shown in Figure 1. The phantom includes individually constructed teeth and removable mandible, maxilla, and tongue modules. Soft tissues were constructed of a urethane‐based resin. All pieces are assembled using a base plate with four fixation screws.

**FIGURE 2:**
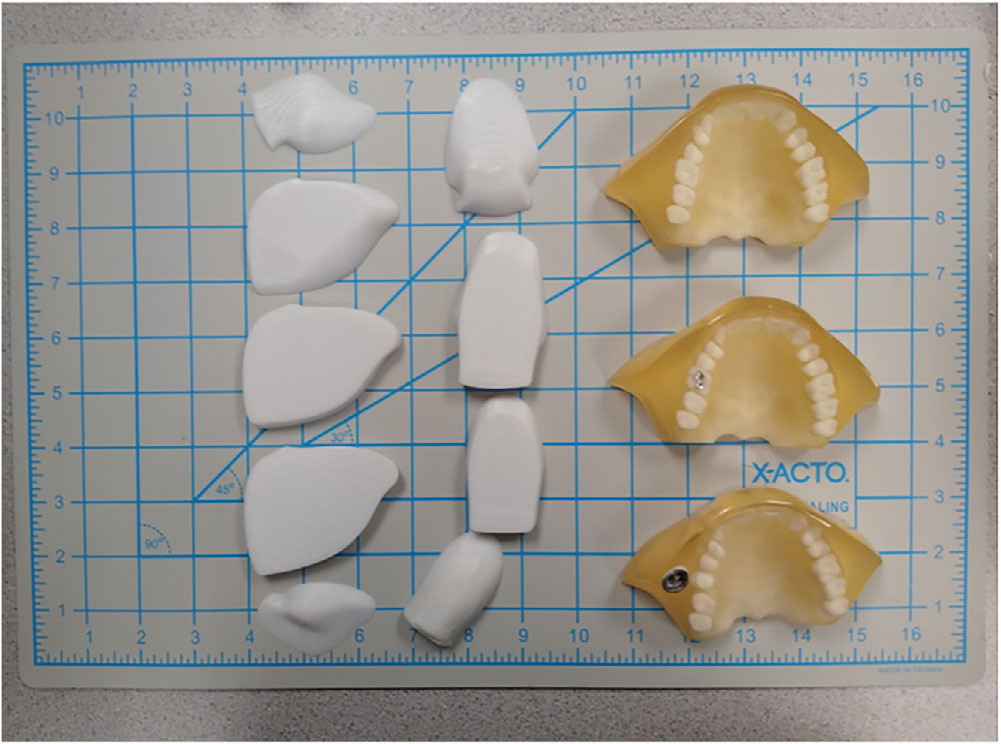
Photograph of modified tongue modules in sagittal (left) and axial (center) orientations as well as maxilla modules (right) depicting control (top), Am (middle), and PFM (bottom) cases.

To test the effect of dental fixtures on treatment delivery, three maxilla modules were used (Figure 2). The first was a control module, where none of the phantom teeth were modified. The second and third modules were modified such that the right first molar in each was implanted with either a 1.5 mm depth central groove occlusal amalgam^17^, ^18^, ^19^, ^20^ (Am) or a porcelain‐fused‐to‐metal (PFM) crown.^21^ The Am was approximately 0.5 × 0.7 cm^2^ in the axial direction and 1.5 mm in thickness, while the PFM crown was approximately 1 × 1 cm^2^ in the axial direction and 0.4 cm in thickness. The amalgam was determined by consult with the local prosthodontics team as the smallest Am fixture likely to be observed in the present patient population (to produce the smallest perturbative effect). The base metal used for the PFM crown was selected based on availability and on consult as being commonly used by the institutional prosthodontics department and confirmed by literature search.^22^

### 2.2 CT simulation and treatment

The phantom was imaged on a clinical Siemens Somatom Definition (Siemens Healthineers, Erlangen, Germany) computed tomography (CT) scanner. The PH‐47 was set up atop a Klarity Cushion (Klarity Medical Products, Heath, OH, USA) and a three‐point Orfit mask (Orfit Industries, Wijnegem, Belgium). The standard treatment planning scanning protocol (120 kVp) for head and neck (H&N) patients was used and reconstructed to 1 mm slices. In‐plane pixel size was 1.3 × 1.3 mm^2^.

Scans including Am and PFM fixtures were reconstructed to an extended dynamic range with metal artifact reduction. Extended dynamic range reconstructions allow the mitigation of intensity saturation by high Z materials at the expense of CT number precision. Am and PFM features were contoured on these scans and overridden to Hounsfield unit (HU) values associated with relative stopping powers (S_rel_) equal to previously measured values for experimental amalgams and base metals (4.985 and 5.400, respectively).^23^ Structure sets were otherwise identical to those used in the control plan.

These scans were rigidly registered to the scans without fixture materials using the software included in the institutional treatment planning system (TPS). All dose calculations were performed on rigidly registered scans without fixture materials, employing material overrides for Am and PFM fixtures where appropriate as contoured from Am and PFM scans. Contouring of the phantom was performed by a physician member of the institutional H&N treatment group, including clinical target volumes (CTV) and organs at risk (OAR) typical of a base‐of‐tongue (BoT) treatment. For simplicity of modelling and measurement, a homogeneous, 54 Gy target volume was considered for planning. Planning was performed on the reconstructed volume without implanted fixtures using the proton convolution superposition (PCS) algorithm (v.15.6.06) in Eclipse v.15.6 (Varian Medical Systems, Palo Alto, CA, USA). Dose grid resolution was 2.5 × 2.5 × 1.0 mm^3^.

Treatment was delivered using our standard H&N image‐guided workflow, with anterior oblique beams being delivered with couch angles of 0 and 180 degrees, respectively due to the half‐gantry configuration of the delivery system. The phantom was positioned using the same immobilization devices used for simulation on a H&N couch top. Image guidance was performed with orthogonal oblique stereoscopic x‐rays. Shifts and rotations were optimized to minimize disagreement between x‐ray images and automatically generated digitally reconstructed radiographs (DRRs) at the setup couch position. Phantom position was radiographically verified at each treatment couch angle.

### 2.3 Dosimetry

Dosimetric measurements were performed using three‐dimensional (3D) printed custom tongue modules. Each 3D printed tongue module used the same polycarbonate material that was designed and commissioned for use in a proton range shifting device.^24^

### 2.4 Gafchromic film

Film measurements were executed with custom 3D printed tongue modules (Figure 2). Tongues were printed in sagittal (left) and axial slices (center). EBT‐3 film was cut to fit between slices. All films for a given orientation (i.e., sagittal or axial) were exposed simultaneously. Placement of axial pieces of film is depicted by the horizontal contours seen in the right panel of Figure 1. Films were scanned at a resolution of 72 dpi (i.e., 0.35 mm/pixel).

Dosimetric calibration for inter‐measurement comparison was performed by delivering known doses (0, 50, 100, 150, 200, 250, 300, 350, 400 cGy) to square strips of EBT‐3 film using a linac operating at 6 MV. Film calibrations were performed using a linear accelerator due to the LET quenching effect observed when using proton beams.^25^, ^26^ Intensities were logarithmically transformed, yielding optical density (OD). The square of the OD was found to be linear as a function of delivered dose and a first‐order least‐squares polynomial regression was executed to yield the calibration curve. Each film was converted into a dose map according to the empirical measurement of the calibration curve.

### 2.5 Experimental design

Experimentally, a series of potential scenarios were considered: 1) a control plan with no fixture overrides; 2) plans where fixture overrides were included for dose optimization. In scenario 1, measurements without and with dental fixtures (i.e., Am and PFM) were executed. For scenario 2, a measurement was acquired with the maxilla insert corresponding to the overridden fixture in the plan (e.g., measurement using the Am maxilla was acquired for the plan with Am overrides). A series of BoT plans were created with the goal of delivering a uniform 54 Gy dose to the CTV over 30 fractions. Individual 1.8 Gy fractions were delivered in each scenario.

### 2.6 Treatment planning

All tested plans were based on a clinically representative treatment geometry for patients without dental fixtures, employing two anterior oblique beams (± 45 degrees with respect to patient midline) and a posterior beam. Typical standard of practice (SOP) suggests using a multi‐field optimization (MFO) approach for H&N plans to better avoid OARs while maximizing target coverage. Further, given that MFO planning is unconstrained by field‐to‐field homogeneity as is the case with single‐field optimized (SFO) plans, the former technique was exclusively used for the study to determine the extent to which the optimizer is able to compensate for implants. In all cases, the plan was robustly optimized for up to 3 mm shifts in the cardinal directions (e.g., L/R, A/P, and S/I) and 3% range uncertainty. Optimization objectives were the same for each scenario and are tabulated in Table 1.

**TABLE 1:** Optimization objectives for all tested scenarios given a 54 Gy prescription dose.

Structure	Metric	Target quantity	Priority
CTV	V100%	100%	100
CTV	V55Gy	0%	96
Brainstem	V100%	0%	75
Spinal cord	V43Gy	0%	83
Larynx	Mean	<30 Gy	75
Left parotid	Mean	<19 Gy	79
Right parotid	Mean	<19 Gy	75

### 2.7 Monte Carlo simulation

Measurements and TPS plans were compared to a previously validated TOPAS Monte Carlo (MC)^27^ simulation of our proton treatment system (Hitachi ProBeat V, Tokyo, JP).^28^ CT image volumes, structure sets, treatment plans, and dose volumes were generated according to section 2.6 and exported from the TPS. The MC simulation used these exported data to recalculate delivered dose for all cases outlined in Table 2. Fixture materials in each case were overridden based on previously found elemental mass proportions.^29^ All simulations were performed using 10^8^ protons.

**TABLE 2:** Per‐field and total MU for each treatment plans without and with either Am or PFM dental fixture overrides.

	Gross MU				Fractional MU			
Override	RAO	Post	LAO	Total	RAO	Post	LAO	Total
None	61.93	66.10	58.98	187.01	0.331	0.354	0.315	1.000
Am	64.16	63.46	59.09	186.71	0.344	0.340	0.317	1.001
PFM	63.65	62.62	60.11	186.38	0.342	0.336	0.323	1.001

## 3 RESULTS

### 3.1 Comparison of treatment plans

**FIGURE 3:**
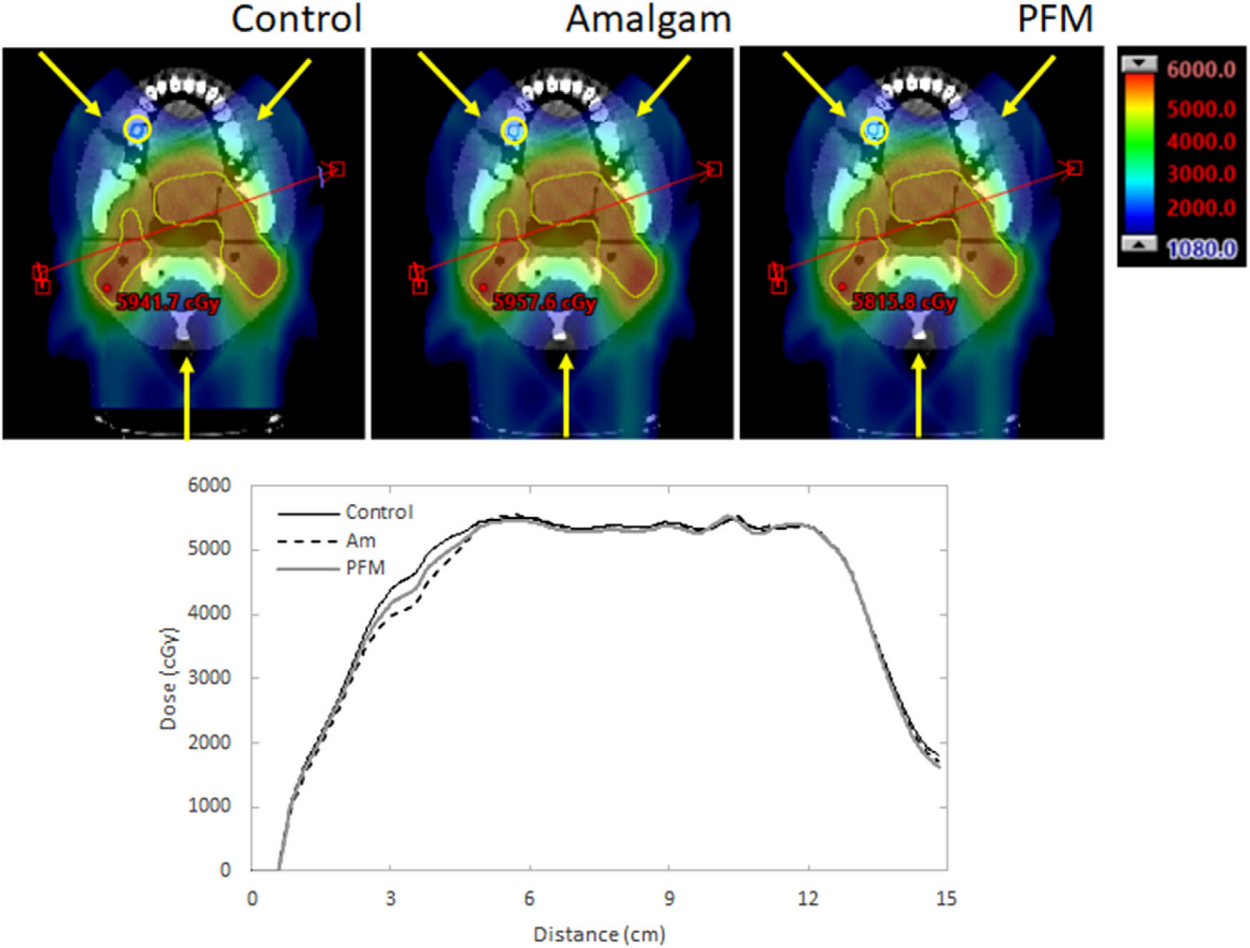
Comparison of treatment plans for the control case (e.g., no implants; left), Am filling (center), and PFM crown (right) along with a comparison of dose profiles (bottom) of a line drawn through the depicted red arrow. Filling and crown plans were optimized using appropriate material overrides. The affected tooth is circled and the beam directions are indicated by the arrows. This equivalency is reinforced in the bottom of Figure 3, which plots comparisons of equivalent dose profiles, drawn through the tongue module. While some differences are observable at the periphery of the profiles, uniform doses of approximately 54 Gy were largely achieved.

Figure 3 plots comparisons of calculated dose planes output by the TPS for each variation of the treatment plan along with comparisons of dose profiles (bottom) for a line drawn through the irradiated area (depicted by the red arrow). Dose planes depicted in the second and third columns correspond to plans optimized with Am and PFM overrides, respectively. The plan in the left column (control) had no overridden values. While some variation exists, visual comparison over the depicted dose display (1080–6000 cGy) suggest qualitative clinical dosimetric equivalence between all plans.

### 3.2 Comparison of beam monitor units

Tabulated in Table 2 are the per‐field monitor units for each plan. Plans distributed slightly more dose to the anterior oblique (AO) beams, exhibiting preferential weighting towards the left (L) AO beam over the right (R). Addition of fixture materials resulted in the optimizer increasing weights towards AO beams for both Am and PFM override plans. Further, increases in dose distribution towards the fixture‐side beam (RAO) were observed Am and PFM override plans, respectively, compared to the LAO beam.

### 3.3 Film measurements

**FIGURE 4:**
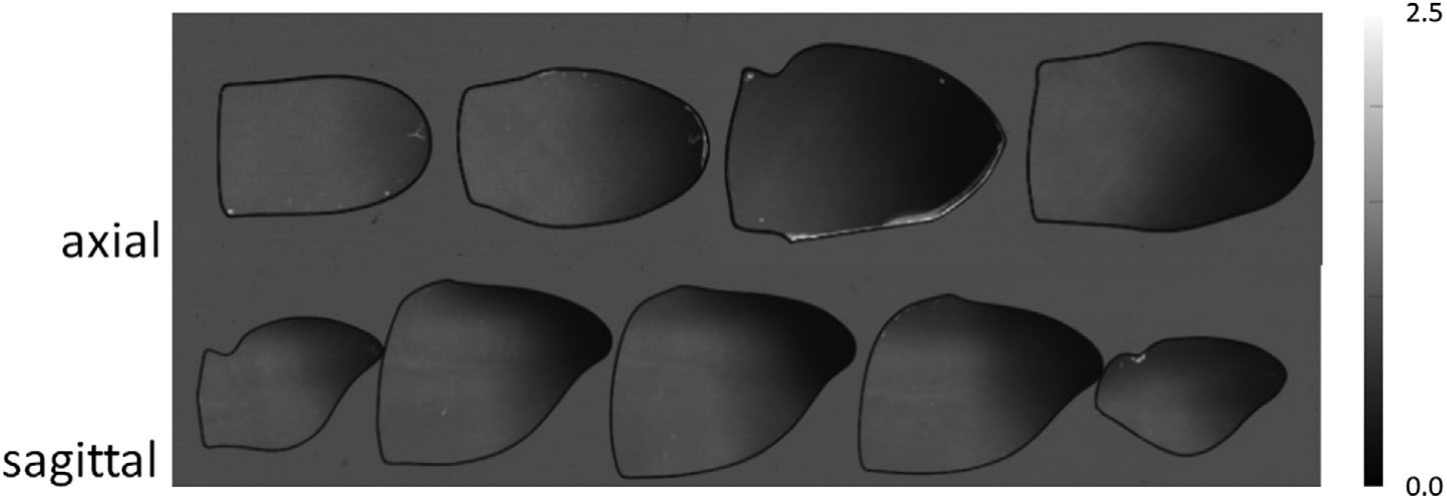
Comparison of film‐measured axial (top) and sagittal (bottom) dose maps for planned BoT treatment. No structures were overridden during optimization and no fixtures were present during treatment delivery.

**FIGURE 5:**
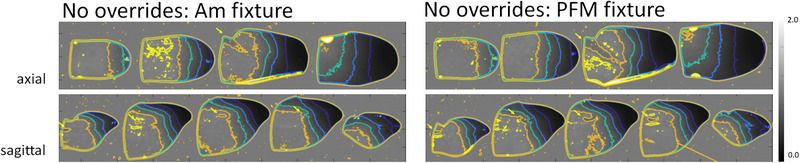
Comparison of film‐measured axial and sagittal dose maps for planned BoT treatment. No structures were overridden during optimization but Am (left) and PFM (right) fixtures were included during treatment delivery. Arrows are included to point out dose shadowing effect. Isodose lines of 30%, 50%, 70%, 90%, and 105% are depicted in indigo, blue, cyan, orange, and yellow, respectively.

**FIGURE 6:**
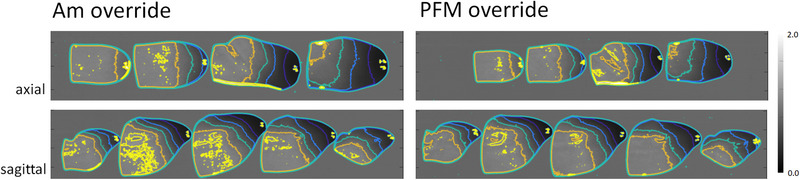
Comparison of film‐measured axial and sagittal dose maps for planned BoT treatment. Am (top) and PFM (bottom) fixtures were overridden for optimization and included during treatment delivery. Isodose lines of 30%, 50%, 70%, 90%, and 105% are depicted in blue.

Figures 4, 5, 6 display axial and sagittal dose maps calibrated according to methods outlined in section 2.4 and normalized to the mean value of the irradiated area of the most inferior axial film slice. Additionally, Figures 5 and 6 depict isodose lines of 30%, 50%, 70%, 90%, and 105%. Viewing Figure 4, where plans with no overridden features were delivered to phantoms with no implanted fixtures, a uniform dose delivered to the BoT is confirmed. Delivering the same plan to phantoms with either implanted Am (Figure 5; left) or PFM (Figure 5; right) fixtures result in dose shadowing directly behind the fixture and in the path of the RAO beam. When the Am fixture is not accounted for in treatment planning, a maximum cold spot of 21% is observed. When PFM fixtures are not overridden, the maximum observed cold spot was quantified at 13%. Cold spots over the measured points‐of‐interest were found to be roughly uniform within individual sagittal film slices.

Figure 6 displays film‐measured axial and sagittal dose maps for deliveries using Am (left) and PFM (right) overridden plans. While fixtures were overridden and the optimizer adjusted beam weights as reported in section 3.2, dose perturbation by each material is observed in all cases, again, directly in‐line with the RAO beam. Isodose lines of 30%, 50%, 70%, 90%, and 105% are depicted in indigo, blue, cyan, orange, and yellow, respectively.

Relative loss was calculated according to:

(1) $$\mathrm{Relative}\;\mathrm{loss}=\frac{D_{depression}}{D_{flat}}\;*100\%,$$

where *D* is the mean measured dose, at the (*flat*) regions and the local minima (*depression*) created by the dental fixture. D*_flat_* was defined by measuring the mean value of the irradiated region within the most inferior axial film, roughly 1 cm^2^ in size. The D*_depression_* was quantified as the mean value for three central, sagittal films sampled over three points (most posterior, central, and most anterior) within the dose shadow. Relative losses are tabulated in Table 3. Measured losses in all cases ranged from 13%−20%.

**TABLE 3:** Measured relative losses as well as the standard deviation of their quantification behind fixture material for central three sagittal films.

Fixture override	Fixture treated	Relative loss (%)	Standard deviation (%)
None	Am	21.2	9.3
	PFM	20.3	10.4
Am	Am	13.6	9.8
PFM	PFM	13.6	7.3

### 3.4 Monte Carlo simulation

**FIGURE 7:**
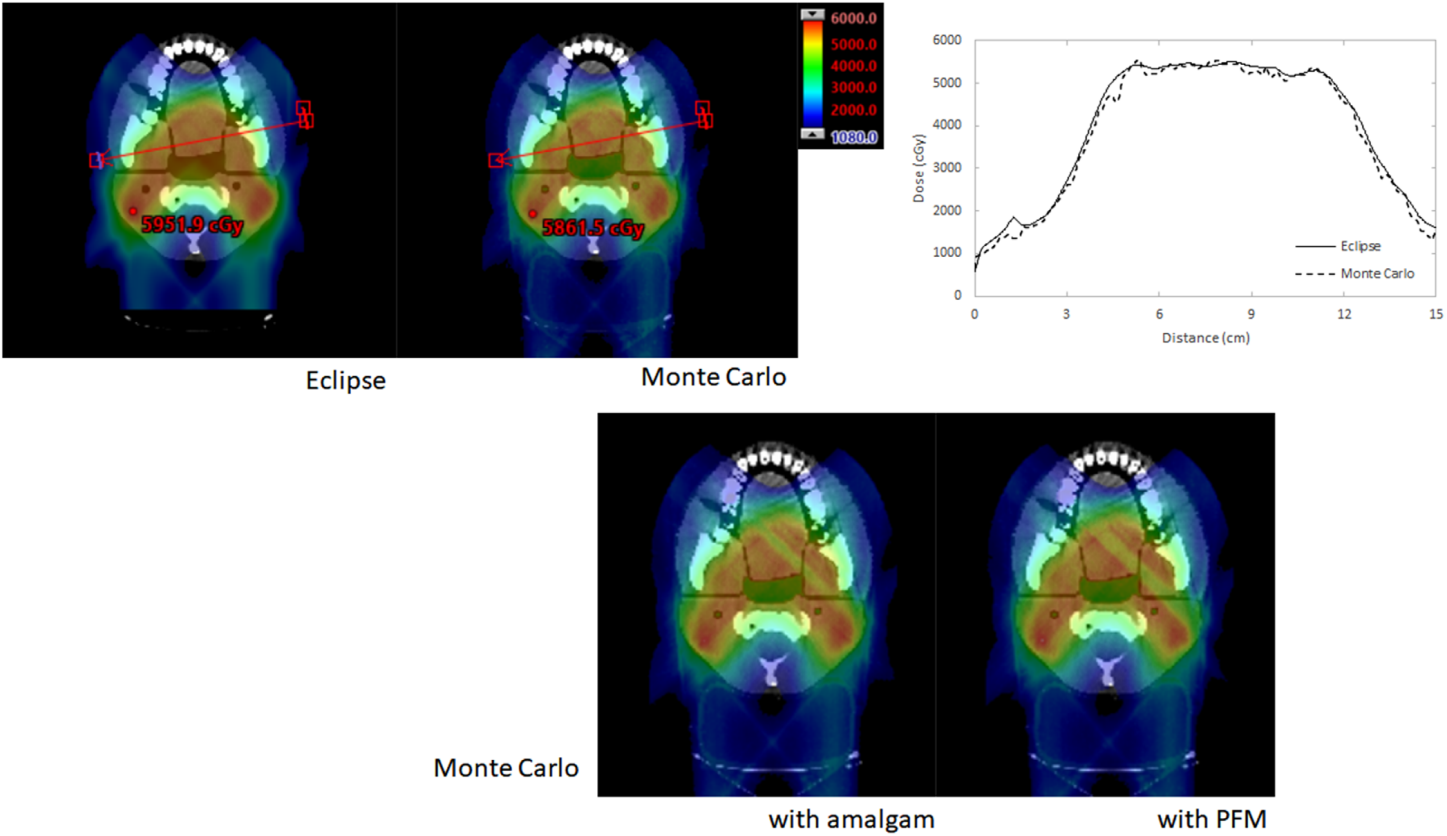
Comparison of Eclipse (top left) and Monte Carlo (top center) dose calculations for plan without material overrides and plot of dose profiles (top right) drawn through depicted arrow. Monte Carlo dose calculations may be seen in the bottom row for the same plan delivered to a phantom with implanted amalgam (left) and PFM crown (right).

Figure 7 depicts dose planes for the treatment plan without overridden fixtures generated in Eclipse (top left). It is accompanied by Monte Carlo simulated dose planes without (top center) and with implanted (Am: bottom left, PFM: bottom right) fixtures. A comparison of the dose profile (top right) drawn through the base‐of‐tongue for Eclipse and Monte Carlo calculations of the plan without fixtures is also included. In simulating delivery of the intended treatment plan without overrides through a patient with Am or PFM fixtures, comparing the point of maximum deviation yields losses of 17% and 14%, respectively.

**FIGURE 8:**
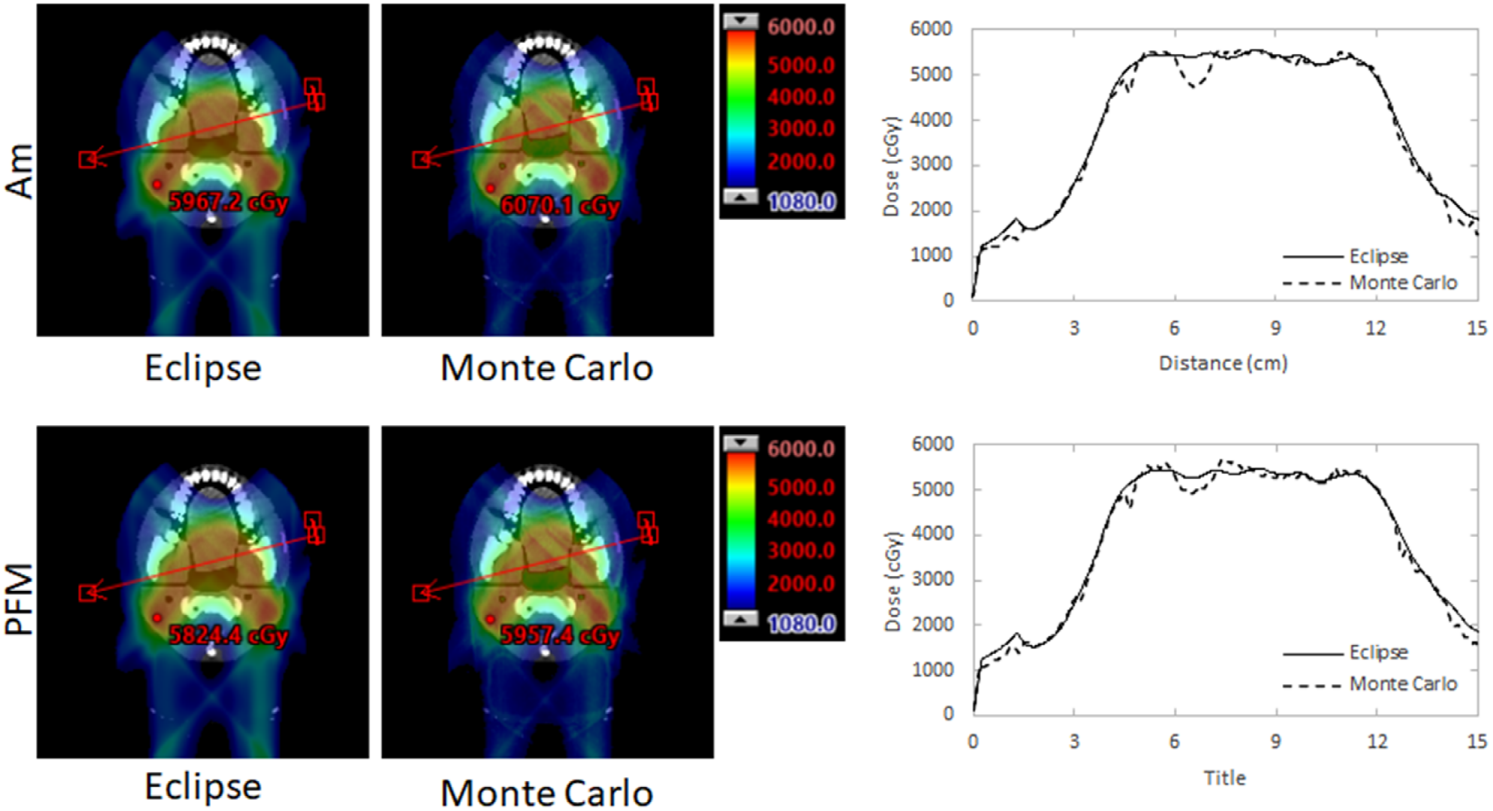
Comparison of Eclipse and Monte Carlo calculated dose planes (left) and profiles (right) drawn through the depicted arrow for Eclipse‐generated Am (top) and PFM (bottom) overridden plans.

Figure 8 depicts TPS and MC calculations for a single dose plane of the tested plans including Am overridden (left group) and PFM overridden (right group) structure sets. While the TPS calculation of the plan depicts little residual dose shadowing effect, the MC simulation clearly displays losses in dose directly behind the implanted fixture in the direction of the RAO beam. A comparison of the mean doses measured along the path of the beam behind the dental fixture at the plane of maximum deviation indicates losses of to 10% and 8% for Am‐ and PFM‐overridden plans, respectively. Standard deviations for these measurements were observed at 2.2% and 2.5% for MC and Eclipse calculations of the Am plan, respectively, and 1.8% and 2.1% for MC and Eclipse calculations of the PFM plan, respectively.

## 4 DISCUSSION

Clinically equivalent treatment plans were created for the control, Am filling, and PFM crown scenarios (Figure 3). According to comparisons of planned MU, as expected per clinical experience with SOP plans, a small sum of additional weight was allocated towards the AO beams. Further, compensation for installed and overridden fixtures resulted in slightly greater beam weight allocated towards the beam from the affected side (RAO).

Addition of Am and PFM materials produced a dose shadowing effect in the same direction as the affected (RAO) beam when unaccounted for in the treatment plan. As observed in Table 3, film measurements quantify the effect from 13% to 21%. According to film measurements, without compensation, this shadow effect results in cold spots of 21% and 20% in magnitude behind Am and PFM fixtures, respectively. Good agreement between Monte Carlo simulations and TPS dose calculations was confirmed for plans without overridden dental materials. Plans generated by the TPS including fixture overrides appeared to resolve cold spots arising from the dose shadowing phenomenon (Figure 3). However, upon measurement (depicted in Figures 4, 5, 6) residual cold spots were observed in both film and MC simulation (Figure 8) for both cases. Disagreement between TPS and MC for Am and PFM override plans were observed to be 11% and 9% respectively due to the residual cold spots. Disagreements in quantification of the shadowing effect between measurement and MC simulation may be the result of uncertainties in the physical film measurements and actual location of the film with respect to the implant. EBT‐3 film has been noted to quench in response to high LET conditions.^25^, ^26^ This under‐response is difficult to estimate given the complexity of treatment conditions, involving both intensity and energy modulation. Its effect in pristine Bragg peaks has been quantified as low as 10% and as high as greater than 20%.^25^, ^26^ Further, because the materials used to construct the PH‐47 phantom are not precisely known or quantified, uncertainties in relative stopping power and range of protons within the phantom are unaccounted for.

While overriding Am and PFM fixtures with HU values associated with S_rel_ reduces the severity of cold spots, the TPS underestimates the perturbative effect, resulting in inadequate compensation and incorrect dosimetric representation in comparison to measurement and MC simulation. Sources of TPS disagreements are currently the subject of ongoing studies. As such, treatment of patients with dental fixtures without employing avoidance strategies may result in large dosimetric errors in delivery. Continued work on development and assessment of Monte Carlo optimization and implementation of more sophisticated dose kernels may help to mitigate these issues.

## 5 CONCLUSION

Inclusion of dental fixtures perturbs proton treatment delivery in the form of a dose shadow effect in the direction of the affected beam. Inclusion and override of these fixtures to CT numbers relating to measured S_rel_ values does not entirely mitigate the dose shadowing effect in our tests. As such, dose calculations for deliveries through these materials must be confirmed either through empirical measurement or Monte Carlo simulation. In lieu of Monte Carlo optimization or more accurate modeling algorithms, best practices of avoiding delivery through these fixtures remain.

## AUTHORS CONTRIBUTION

All authors materially contributed to the experimental design, analysis, and drafting of the submitted manuscript. Principle data collection and analysis were performed by Yue‐Houng Hu and Daniel W. Mundy. Yue‐Houng Hu was primary in composition of the manuscript. All authors substantially contributed to the editing and interpretation of the content.

## CONFLICT OF INTEREST STATEMENT

The authors declare no conflicts of interest.
